# Supplementary material for: Climate-induced range shifts drive adaptive response via spatio-temporal sieving of alleles
Source: Nat Commun. 2023 Feb 25;14:1080. doi: 10.1038/s41467-023-36631-9 (PMC9968346; doi:10.1038/s41467-023-36631-9)
Supplement: Supplementary file 3 — Description of Additional Supplementary Files [file 41467_2023_36631_MOESM3_ESM.pdf]

Description of Additional Supplementary Files

File Name: Supplementary Data 1
Description: **Sampling of wild populations.** Population name, taxonomic denomination, geographic coordinates (WGS 84), elevation (meters above sea level), date sampled, country (ISO 3166-1 alpha-2 codes), and number of sequenced individuals per population are reported. Note that taxonomic names adhere to established epithets used in floristic treatments. Revision of the taxonomy of D. sylvestris s.l. is ongoing and will account for the results of this study. The populations used in the following population genetic analyses are reported: PCA (all individuals from all populations; alternative run on 125 individuals per meta-population indicated in parentheses), genetic distance (5 individuals per population), admixture (125 individuals per metapopulation), badMIXTURE (22 and 65 individuals per meta-population, for the Apennine-Balkan and Alpine-Balkan analyses respectively), diversity (EEMS; 5 individuals per population), ψ (10 individuals per population), demography (20 individuals per population), and gradient forest (GF; 14 individuals per population). For populations used in GF, superscripts "*" and "†" denote the subset of populations used in the genetic distance tree, PCA and presenceabsence Venn diagram of the top 1000 environmentally-associated SNPs, and used to calculate population genetic diversity and neutrality statistics (geographically proximate population-pairs), respectively.
