# Supplementary material for: Climate-induced range shifts drive adaptive response via spatio-temporal sieving of alleles
Source: Nat Commun. 2023 Feb 25;14:1080. doi: 10.1038/s41467-023-36631-9 (PMC9968346; doi:10.1038/s41467-023-36631-9)
Supplement: Supplementary file 5 — Reporting Summary [file 41467_2023_36631_MOESM5_ESM.pdf]

nature portfolio

Corresponding author(s): Hirzi Luqman
Simone Fior
Alex Widmer

Last updated by author(s): Feb 2, 2022

Reporting Summary

Nature Portfolio wishes to improve the reproducibility of the work that we publish. This form provides structure for consistency and transparency in reporting. For further information on Nature Portfolio policies, see our Editorial Policies and the Editorial Policy Checklist.

Statistics

For all statistical analyses, confirm that the following items are present in the figure legend, table legend, main text, or Methods section.

n/a	Confirmed	
☐	☒	The exact sample size (*n*) for each experimental group/condition, given as a discrete number and unit of measurement
☐	☒	A statement on whether measurements were taken from distinct samples or whether the same sample was measured repeatedly
☐	☒	The statistical test(s) used AND whether they are one- or two-sided<br>*Only common tests should be described solely by name; describe more complex techniques in the Methods section.*
☐	☒	A description of all covariates tested
☐	☒	A description of any assumptions or corrections, such as tests of normality and adjustment for multiple comparisons
☐	☒	A full description of the statistical parameters including central tendency (e.g. means) or other basic estimates (e.g. regression coefficient) AND variation (e.g. standard deviation) or associated estimates of uncertainty (e.g. confidence intervals)
☐	☒	For null hypothesis testing, the test statistic (e.g. *F*, *t*, *r*) with confidence intervals, effect sizes, degrees of freedom and *P* value noted<br>*Give P values as exact values whenever suitable.*
☐	☒	For Bayesian analysis, information on the choice of priors and Markov chain Monte Carlo settings
☐	☒	For hierarchical and complex designs, identification of the appropriate level for tests and full reporting of outcomes
☐	☒	Estimates of effect sizes (e.g. Cohen's *d*, Pearson's *r*), indicating how they were calculated

Our web collection on statistics for biologists contains articles on many of the points above.

Software and code

Policy information about availability of computer code

Data collection	No software was used in data collection. Genomic data was produced as detailed in Methods and Supplementary Method S1.1.
Data analysis	Code used for performing demographic inference, running distribution models, visualizing shifts in environmental space, performing gradient forest and calculating glacial genomic offsets is available at our Github repository (https://github.com/hirzi/RhEA) with associated DOI (https://doi.org/10.5281/zenodo.7581797).<br><br>In addition, we used the following third-party software for data analysis: ANGSD version 0.933 , ASTRAL-III version 5.6.3, ATLAS versions 0.9 and 1.0, bamUtil version 1.0.14, bcftools version 1.8, Beagle version 3.3.2, BWA version 0.7.17, EEMS, FastME version 2.1.6.1, freebayes version 1.3.1, GATK version 3.5, MAKER version 2.31.5, MixPainter, moments version 1.0.0, ngsTools version 1.0.1, PCAngsd version 0.98, PHAST version 1.4, Picard Toolkit version 2.0.1, Sambamba version 0.6.8, SAMtools version 1.8, Trimmomatic version 0.35, vcflib popStats version 1.0.1.1, adegenet R package version 2.1.1, adespatial R package version 0.3.8, akima R package version 0.6.2, badMIXTURE R package version 0.0.0.9000, dbscan R package version 1.1.2, dismo R package version 1.1.4, ecospat R package version 3.0, extendedForest R package version 1.6.1, FNN R package version 1.1.2.1, gdistance R package version 1.2.2, ggplot2 R package version 3.3.2, gradientForest R package version 0.1.18, ipdw R package version 0.2.6, randomForest R package version 4.6.14, rangeExpansion R package version 0.0.0.9000, usdm R package version 1.1.18. If no version number is provided above, this is because no version number was associated and provided with the software.<br><br>Usage of all listed software and code is detailed in Methods and Supplementary Methods.

For manuscripts utilizing custom algorithms or software that are central to the research but not yet described in published literature, software must be made available to editors and reviewers. We strongly encourage code deposition in a community repository (e.g. GitHub). See the Nature Portfolio guidelines for submitting code & software for further information.

Data

Policy information about availability of data

All manuscripts must include a data availability statement. This statement should provide the following information, where applicable:

- Accession codes, unique identifiers, or web links for publicly available datasets
- A description of any restrictions on data availability
- For clinical datasets or third party data, please ensure that the statement adheres to our policy

Raw sequencing reads for the 1261 low-coverage D. sylvestris whole genomes are deposited and available at the European Nucleotide Archive (ENA) under accession code PRJEB53522 (www.ebi.ac.uk/ena/data/view/PRJEB53522). Raw sequencing reads for the across-species dataset are available at ENA under accession code PRJEB54098 (www.ebi.ac.uk/ena/data/view/PRJEB54098). The D. sylvestris genome reference assembly and structural annotation are available at the Dryad repository: https://doi.org/10.5061/dryad.x0k6djhng. Species occurrence data is available at the GitHub repository: https://github.com/hirzi/RhEA (https://doi.org/10.5281/zenodo.7581797). Sample accessions and metadata are provided in Supplementary Data 1.

Environmental data used was downloaded from CHELSA (version 1.2; https://chelsa-climate.org) and SoilGrids (2020 version; https://soilgrids.org). Topographic data was downloaded from CHELSA and GMTED2010 (2010 version; https://topotools.cr.usgs.gov/gmted_viewer/gmted2010_global_grids.php).

Species occurrence data was acquired from Conservatoire Botanique National Méditerranéen de Porquerolles (CBNMed; http://flore.silene.eu), Conservatoire Botanique National Alpin (CBNA; http://flore.silene.eu), GBIF (https://www.gbif.org; https://doi.org/10.15468/dd.zzqdys), iNaturalist (https://www.inaturalist.org), Info Flora (https://www.infoflora.ch), Wikiplantbase #Italia (http://bot.biologia.unipi.it/wpb/italia), Sweden's Virtual Herbarium (http://herbarium.emg.umu.se), Virtual Herbaria Austria (https://www.jacq.org) and personal collaborators in 2017. This data is deposited in the Github repository (https://github.com/hirzi/RhEA; https://doi.org/10.5281/zenodo.7581797).

Human research participants

Policy information about studies involving human research participants and Sex and Gender in Research.

Reporting on sex and gender	Not applicable - no human research participants are included in this study.
Population characteristics	Not applicable - no human research participants are included in this study.
Recruitment	Not applicable - no human research participants are included in this study.
Ethics oversight	Not applicable - no human research participants are included in this study.

Note that full information on the approval of the study protocol must also be provided in the manuscript.

Field-specific reporting

Please select the one below that is the best fit for your research. If you are not sure, read the appropriate sections before making your selection.

☐ Life sciences ☐ Behavioural & social sciences ☒ Ecological, evolutionary & environmental sciences

For a reference copy of the document with all sections, see nature.com/documents/nr-reporting-summary-flat.pdf

Ecological, evolutionary & environmental sciences study design

All studies must disclose on these points even when the disclosure is negative.

Study description	This study integrates population genetics with gene-environment models to jointly assess the demographic and adaptive history of Dianthus sylvestris, as shaped by past Quaternary climate fluctuations.
Research sample	A broad sampling of natural populations of Dianthus sylvestris across the species' range.
Sampling strategy	170 natural populations of Dianthus sylvestris were sampled between 2008-2018 for leaf material, which were dried and stored in silica gel. Of these, we selected 116 populations that covered the geographic range and ecological diversity of the species, and 5-20 individuals per population, for DNA extraction and whole-genome sequencing (see sample map in Figure 1 and Supplementary Methods S1.1). This sampling, totaling 1261 individuals, represents the most comprehensive sampling of this species to date.
Data collection	Genomic data was produced from collected leaf material as detailed in Methods and Supplementary Method S1.1.
Timing and spatial scale	Populations were sampled yearly in the summer months (June-September) between 2015-2017. In addition, samples from older collections sampled in the summer months of 2008, 2011 and 2012, and from the winter of 2018, were included. Exact collection dates for all samples are detailed in Supplementary Data 1. The span of sampling (from June 2008 - 2018) was to allow for comprehensive geographic sampling of the species, which required multiple years given the available resources. This span of time,

and collection across different months of the year, are not expected to effect the results of this study (inferences of demography and phylogeography), as these inferences are based on DNA polymorphisms which shift negligibly under such short time-scales and seasonality.

Populations were sampled across the European Alps, the Apennines and the Dinaric Alps. Each geographic location (population) was sampled once (i.e. no longitudinal samples). Sampling dates and geographic coordinates are detailed in Supplementary Data 1.

Data exclusions	See Supplementary Data 1 for the data subsets used for the various analyses.
Reproducibility	Findings were reproduced and supported by multiple, independenent approaches, as reported in the manuscript. Specifically, genetic structure (describing three distinct evolutionary lineages) was supported by principle component analysis, admixture analysis and genetic distance trees. The inference of glacial refugia was supported by clines in genetic diversity, the directionality statistic (psi), and hindcasts of species distribution models. Predictions based on glacial genomic offsets were corroborated by population genetic signatures of wild, contemporary populations.
Randomization	Two levels of hierarchy (natural group allocation) are relevant to this study. First, samples as they belong to a population. This is geographically defined such that all individuals sampled in a locality are considered as belonging to the same population. Two, samples and populations as they belong to a genetic cluster (evolutionary lineage). This was inferred via principle component analysis, admixture profiles and distance trees based on whole-genome data. Population structure was accounted in all analyses.
Blinding	This is not relevant to our study as it does not include an experimental treatment.

Did the study involve field work? ☒ Yes ☐ No

Field work, collection and transport

Field conditions	Field work (sampling of wild populations) was predominantly conducted in the summer months (June-September) in the European Alps, the Apennines and the Dinaridic Alps, and was characterised by typical summer climate in these regions. Three populations were sampled in Italy in winter (January 2018), during mild conditions typical of the Ligurian (Mediterranean) coast during winter.
Location	Locations of sampled populations are given in Supplementary Data 1.
Access & import/export	Access, collection and import/export of samples were conducted in a responsible manner and in compliance with all relevant local, national and international laws. All necessary sampling permits were obtained prior to sample collection. This comprised of sampling permits from Comunità della Vallagarina for sampling in Monte Baldo, Italy (issued on 13 July 2017, valid for the year 2017); from Parco Nazionale Gran Paradiso for sampling in Gran Paradiso National Park, Italy (issued on 12 May 2017, valid for the summer of 2017); from Amt für Natur und Umwelt for sampling in Graubünden, Switzerland (issued on 21 April 2017, valid for the years 2017 and 2018); from Amt für Natur, Jagd und Fischerei for sampling in St. Gallen, Switzerland (issued on 29 May 2017, valid from the date of issue until 31 December 2018), and from Amt für Wald und Landschaft for sampling in Obwalden, Switzerland (issued on 29 May 2017, valid for May-September 2017 and May-September 2018). Nagoya Protocol on Access and Benefit Sharing was followed in countries where it had been ratified at the time of sampling. Dianthus sylvestris is not listed under the Convention on International Trade in Endangered Species of Wild Fauna and Flora (CITES) or under the International Union for Conservation of Nature (IUCN) Red List of Threatened Species.
Disturbance	Minimal disturbance was caused by this study as only a few individuals (5-20) per population were sampled for leaves, and the amount of leaf material taken per plant (15-30 small leaves) was generally a fraction of the plant's total leaf mass.

Reporting for specific materials, systems and methods

We require information from authors about some types of materials, experimental systems and methods used in many studies. Here, indicate whether each material, system or method listed is relevant to your study. If you are not sure if a list item applies to your research, read the appropriate section before selecting a response.

Materials & experimental systems

n/a	Involved in the study
☒	☐ Antibodies
☒	☐ Eukaryotic cell lines
☒	☐ Palaeontology and archaeology
☒	☐ Animals and other organisms
☒	☐ Clinical data
☒	☐ Dual use research of concern

Methods

n/a	Involved in the study
☒	☐ ChIP-seq
☒	☐ Flow cytometry
☒	☐ MRI-based neuroimaging
